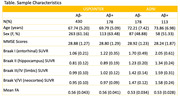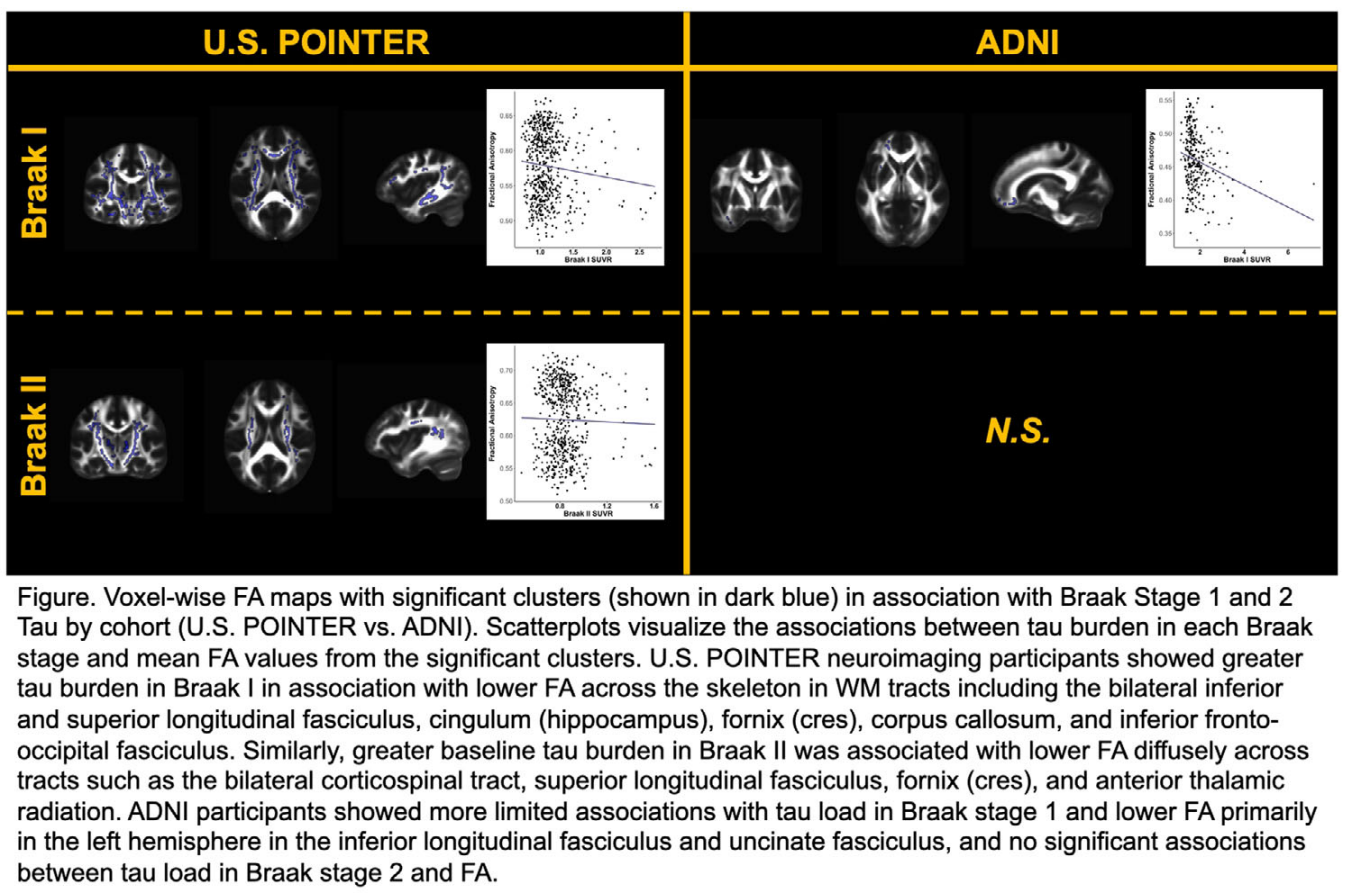# White matter abnormalities in association with amyloid and tau deposition in a diverse cohort with increased risk for Alzheimer’s disease

**DOI:** 10.1002/alz.094009

**Published:** 2025-01-09

**Authors:** Jenna K Blujus, Eliza Rego, Sarah Bica, Kathryn Demos, Athene KW Lee, Meghan C. Riddle, Rena R. Wing, Stephen Salloway, Pauline Maillard, Audrey P. Fan, Charles Decarli, Prashanthi Vemuri, Samuel N. Lockhart, Laura D Baker, Susan M. Landau, Hwamee Oh

**Affiliations:** ^1^ Brown University, Providence, RI USA; ^2^ Butler Hospital, Providence, RI USA; ^3^ Miriam Hospital, Providence, RI USA; ^4^ Alzheimer’s Disease Research Center, University of California Davis, Sacramento, CA USA; ^5^ University of California Davis, Davis, CA USA; ^6^ Mayo Clinic, Rochester, MN USA; ^7^ Wake Forest University School of Medicine, Winston‐Salem, NC USA; ^8^ Wake Forest University, Winston‐Salem, NC USA; ^9^ University of California, Berkeley, Berkeley, CA USA

## Abstract

**Background:**

Neuritic plaques with fibrillar beta‐amyloid (Aß) peptides and tau‐protein neurofibrillary tangles, hallmark features of Alzheimer’s disease (AD) pathology, have been concomitantly associated with white matter (WM) integrity loss, while a unique effect of each pathology on WM integrity in a more demographically diverse population remains unknown.

**Method:**

To examine the degree to which each pathology affects WM integrity in a more diverse non‐demented cohort, Aß and tau PET, diffusion‐weighted imaging (DWI), and cognition (memory and executive function composites) were examined from the U.S. POINTER neuroimaging participants (N = 608; age = 68.34±5.2; 61.8% females; 29% Aß+) and a subset of Alzheimer’s Disease Neuroimaging Initiative (ADNI) participants (N = 291; age = 72.85±7.3; 50% females; 39% Aß+) (Table). Aß‐positivity was determined using tracer‐specific standardized cut‐offs. Tau SUVR was estimated for each Braak stage. For WM integrity, voxel‐based GLMs were applied to FSL‐processed fractional anisotropy (FA) maps using Braak stage tau SUVR and Aß‐positivity status, along with covariates of age, sex, and b‐value profiles.

**Result:**

In U.S. POINTER, independent from Aß‐positivity status, greater tau burden in Braak stages I and II was related to lower FA across the tracts including the superior longitudinal fasciculus, fornix (cres), corpus callosum, and anterior thalamic radiation (Figure). Average FA from supra‐threshold clusters for Braak I and II significantly predicted executive function but not episodic memory. No association between FA and tau burden in Braak stages III/IV or V/VI was found. In the ADNI sample, greater tau burden in Braak I was related to lower FA mostly in left inferior longitudinal fasciculus and left uncinate fasciculus, while negative associations with FA became more diffuse with tau load in Braak III/IV and V/VI. Average FA from supra‐threshold clusters significantly predicted both episodic memory and executive function scores. Across both samples, after accounting for tau burden in each Braak stage, there were no association between Aß‐positivity status and WM integrity.

**Conclusion:**

Distinct associations between tau burden, WM integrity, and cognition were found across the studies, indicating greater impact of early tau burden on WM integrity in U.S. POINTER, accounting for Aß‐positivity status. Factors contributing to the distinct associations across studies need further investigation.